# Machine learning methods to predict leprosy misdiagnosis in Yunnan Province, People's Republic of China

**DOI:** 10.3389/fpubh.2026.1785606

**Published:** 2026-05-08

**Authors:** Yingwu Guo, Lijiao Yin, Hailong Yang, Xi Yang, Xiufeng Yu, Chunyu Zhang, Lijuan Zhou, Fushuai Zhao, Shengqing Lu, Qiaojing He, Lin Han, Weiwei Wang, Youhong Liu, Yu-Ye Li

**Affiliations:** 1 Department of Dermatology Venereology, First Affiliated Hospital of Kunming Medical University, Kunming, China; 2 Wenshan Prefecture Dermatology Prevention and Treatment Institute (Wenshan Prefecture Dermatology Specialist Hospital), Wenshan, Yunnan, China; 3 School of Basic Medical Sciences, Kunming Medical University, Kunming, Yunnan, China

**Keywords:** China, leprosy, machine learning, misdiagnoses, prediction

## Abstract

**Background and objective:**

Leprosy is a chronic infectious disease caused by *Mycobacterium leprae* that is often misdiagnosed. This study aimed to identify factors associated with leprosy misdiagnosis and develop and compare machine learning (ML) models to predict the risk of misdiagnosis.

**Methods:**

A retrospective analysis was conducted on clinical and epidemiological data in 486 diagnosed leprosy patients. The outcome was a binary variable to indicate whether a patient had experienced a prior misdiagnosis. Features analyzed included sociodemographic factors, clinical characteristics, and epidemiological exposures. LASSO regression analysis performed feature selection. Class imbalance was handled using synthetic minority oversampling technique. Nine ML models were trained and validated with a 80–20 data split. The best model performance was evaluated based on AUC-ROC, sensitivity, and specificity. Important features were interpreted using the SHapley Additive exPlanation (SHAP) technique.

**Results:**

Among 486 leprosy patients, 159 (32.7%) experienced misdiagnoses. Nineteen features were selected for model development. The best-performing model was Neural Network, which demonstrated the most balanced performance (AUC: 0.79 and 0.68, sensitivity: 0.93 and 0.78, specificity: 0.68 and 0.57 in train and test, respectively). The SHAP analysis identified key predictors associated with the detection of leprosy misdiagnosis, including mode of detection, aspartate aminotransferase level, gender, and the presence of skin lesions. In addition, ethnicity, education, leprosy reaction, household contact with an active case, and source of infection also contributed to the detection of leprosy misdiagnosis.

**Conclusion:**

Applying ML to clinical data can effectively identify leprosy patients at high risk of being misdiagnosed using clinical, social and epidemiology characteristics. A ML-based support tool could aid frontline healthcare providers to reduce overlooking leprosy diseases.

## 1 Introduction

Leprosy is caused by *Mycobacterium leprae* and remains a global health concern despite decades of control efforts and the availability of multidrug therapy. The World Health Organization reports over 180,000 new cases annually that are concentrated mainly in low- and middle-income countries (1, 2). While global prevalence has declined, the burden of disability, stigma, and continued transmission is sustained by a persistent challenge—misdiagnosis (3, 4).

An accurate diagnosis of leprosy is complicated by its wide clinical spectrum. Skin lesions, sensory impairment, and peripheral nerve involvement can mimic more common dermatological, rheumatological, and neurological conditions (3, 5). Misdiagnoses occur in both high- and low-endemic regions. In endemic countries, limited clinical expertise, stigma, and insufficient diagnostic infrastructure hinder timely recognition (4, 6). In lower-endemic regions such as China, leprosy is often overlooked as physicians less frequently suspect it, which leads to frequent misclassification as dermatitis, psoriasis, neuropathy, or rheumatism (7). These systemic challenges result in delayed treatment, irreversible disability, and ongoing community transmission (3).

Conventional strategies to address misdiagnosis, such as healthcare worker training, community awareness, and improved referral systems, remain essential but insufficient (4). Advances in data science offer new opportunities to complement existing approaches. Machine learning (ML) has the capacity to analyze large, complex datasets and detect subtle patterns in clinical presentations. ML has shown promise in supporting medical diagnosis across infectious, dermatological, and neurological diseases (8). Applied to leprosy, ML models can be trained on clinical, demographic, and epidemiological data to assist frontline providers in differentiating leprosy from its common mimics, even in resource-limited settings where specialist expertise is scarce (9, 10). Importantly, ML approaches can standardize diagnostic support, reduce human error, and potentially shorten the time to diagnosis, thereby mitigating disability and halting transmission (9, 11).

Thus, incorporating ML into leprosy research is a timely and innovative step. It addresses a critical diagnostic gap by offering a scalable, supportive tool for healthcare providers. Our study aims to compare machine learning (ML) models to predict the risk of misdiagnosis of leprosy by using clinical, social and epidemiological data. ML's identification of potential misdiagnosis of leprosy that would be overlooked by human providers would have the potential to transform leprosy control strategies, contribute to earlier detection, reduced disability, and progress toward global elimination goals (9, 11).

## 2 Methods

### 2.1 Study population

We conducted a retrospective cohort study using data between January 2011 and December 2024 from the China Leprosy Management Information System. The system covers all registered leprosy cases, totaling 486 cases in Yunnan Province which have sufficient power to detect misdiagnosis (12).

### 2.2 Outcome

The primary outcome of this study was leprosy misdiagnosis. Misdiagnosis was defined as an incorrect diagnosis rather than leprosy at the time of presentation or during follow-up that was later revised based on clinical, laboratory, or histopathological evidence. This outcome was treated as a binary variable on whether a patient was misdiagnosed with other diseases rather than leprosy (yes) or correctly diagnosed with leprosy (no).

### 2.3 Data management and analysis

The data were managed and analyzed using R version 4.4.2 (The R Foundation for Statistical Computing, Vienna, Austria). The analysis employed various R packages across different stages of the ML workflow. Feature selection utilized glmnet version 4.1–8 (LASSO regression), and randomForest version 3.3.3 (variable importance). Class imbalance was handled using synthetic minority oversampling technique (SMOTE) in caret version 7.0–1. Model performance was assessed using stats version 4.4.2 (LR), rpart version 4.1.23 (DT), randomForest version 3.3.3 (RF), class version 7.3–22 (KNN), e1071 version 1.7–16 (SVM and NB), xgboost version 1.7.8.1 (XGB), gbm 2.2.2 (SGBT), and nnet version 7.3–19 (NNET). Model validation used caret version 7.0–1 and MLmetrics version 1.1.3 (accuracy, precision and F1 score of the model) and pROC/ROCR version 1.18.5 (AUC). The sensitivity and specificity were calculated from confusion matrix derived from actual and predicted values on the risk of misdiagnosis in each model. Model interpretability was assessed using iml version 0.11.4, and shapviz version 0.9.7 (SHAP values).

### 2.4 Machine learning procedures

The ML workflow comprised six main steps: data splitting, feature selection, model development, validation, performance, and identification of important variables.

#### 2.4.1 Data splitting

The dataset was randomly partitioned into recommended 80% for model training and 20% for internal testing to support model development and validation (13).

#### 2.4.2 Feature selection

Feature selection was performed using Least Absolute Shrinkage and Selection Operator (LASSO) regression (L1 penalty) on the 80% training dataset to identify key variables associated with misdiagnosis of leprosy patients (14). It was applied to shrink less informative variables to zero that enabled the selection of relevant predictors. A 10-fold cross-validation was employed, where the training data were divided into 10 subsets. In each round, 9 folds were used for training and 1 for validation, which was repeated across all folds. The optimal regularization parameter (λ) was computed based on the mean cross-validation error and misclassification error to identify relevant predictors. The value λ.min, which minimizes this error, was selected to maximize predictive performance to provide greater accuracy than λ.1se, which is the more regularized alternative within one standard error of the minimum (15).

#### 2.4.3 Handling class imbalance using SMOTE

Our data is likely to be imbalanced with a minority class of misdiagnosis cases and a majority class of leprosy cases. To address class imbalance, the SMOTE was applied to the training dataset. SMOTE generates synthetic minority class samples by interpolating between nearest neighbors in the feature space, thereby improving class balance and model performance. This approach was chosen to enhance minority class detection while minimizing information loss and reducing the risk of overfitting compared with simple duplication methods (16).

#### 2.4.4 Model development and validation in training and test dataset

Model development was conducted in the training set. The SMOTE was implemented within each training fold. All models were developed using a consistent set of key predictors identified through LASSO regression to ensure comparability across different ML approaches. A set of nine machine learning models were employed to construct a predictive model. The models included logistic regression (LR) that predicted the probability of having a misdiagnosis based on a linear relationship with the features.

Tree-based models, such as decision tree (DT), random forest (RF), gradient boosting tree (SGBT), and extreme gradient boosting (XGB), build trees that split the data based on feature values to reveal patterns and associations between features and the misdiagnosis of leprosy. Features that contribute the most to reducing impurity (misclassification) at each split within the decision trees are considered the most influential in predicting the risk of misdiagnosis of leprosy.

The instance-based model, k-nearest neighbors (KNN), which identified patients with similar feature values, tended to have misdiagnosis of leprosy. Support vector machine (SVM) identifies the optimal hyperplane that best separates patients with and without misdiagnosis of leprosy based on their feature values. Features that strongly influence the separation boundary are considered more important in distinguishing between patients with and without misdiagnosis of leprosy.

Naive Bayes (NB), which is a probabilistic model, uses Bayes' theorem and calculates the probability of a misdiagnosis of leprosy given the presence or absence of each feature. NB combines these probabilities to predict the overall risk. Features with stronger conditional probabilities given the outcome are considered more associated with misdiagnosis of leprosy.

Neural network (NNET) captures non-linear relationships through layers of neurons. Through multiple layers of interconnected neurons, the network learns patterns that associate the features and misdiagnosis of leprosy. The strength of association between each feature and the outcome is reflected in the weights assigned to connections of neurons, which allows the model to learn which features are more influential in predicting the risk of developing misdiagnosis of leprosy. Subsequently, the models were evaluated on the remaining 20% of the dataset that was reserved for internal validation.

#### 2.4.5 Model performance

The predictive performance of features (clinical diagnoses and procedures) in all models, using both training and test data, on the risk of leprosy misdiagnosis was comprehensively evaluated using standard classification metrics, which included area under the receiver operating characteristic curve (AUC-ROC), accuracy, sensitivity, specificity, precision, and F1 score (17). The AUC-ROC measures a model's ability to discriminate between patients with and without misdiagnosis of leprosy across all classification predictive thresholds of feature variables. AUC–ROC was interpreted as follows: values < 0.7 indicate poor discrimination, 0.7–0.8 acceptable discrimination, and 0.8–0.9 excellent discrimination (18).

A confusion matrix was used for both train and test datasets. It was constructed using predicted misdiagnosis of leprosy (yes/no) from each model and actual misdiagnosis of leprosy (yes/no) in the train or test data sets. This matrix provided a 2 × 2 classification by tabulating a predicted misdiagnosis and leprosy with the actual misdiagnosis of leprosy. It represented true positives (correctly predicted actual misdiagnosis), true negatives (correctly predicted actual leprosy), false positives (actual leprosy incorrectly predicted as misdiagnosis), and false negatives (actual leprosy incorrectly predicted as misdiagnosis). Accuracy reflected the overall correctness of the model by measuring the proportion of true predictions (both true positive and negative) among the total observations. Sensitivity was the proportion of misdiagnosis that was correctly predicted by the model. Specificity was the proportion of actual leprosy that was correctly predicted by the model. Precision measured the proportion of predicted misdiagnosis that was truly an actual misdiagnosis. A higher F1 score indicated that the model was more effective at correctly identifying a misdiagnosis while minimizing both false positives and false negatives (19). AUC and F1 score have been used to classify whether diseases or non-diseases with imbalanced data (16). Net Benefit compared the number of misdiagnoses correctly predicted by the model with the number of actual leprosy incorrectly predicted as misdiagnosis (20).

The best models were selected based on higher specificity and sensitivity. The sensitivity predicts misdiagnosis of leprosy that can be overlooked by human errors while specificity predicts accurate leprosy (21).

#### 2.4.6 Identification of important features

Model interpretation was performed using SHapley Additive exPlanations (SHAP), which quantified the importance of each feature in the model by calculating the contribution value, known as the Shapley value, for each feature toward the predicted outcome. A higher SHAP value for a variable indicated a greater contribution to the model's prediction. An increase in the feature level (from 0 = no to 1 = yes) could be associated with the risk of misdiagnosis either positively by a positive SHAP value or negatively by a negative SHAP value (22).

## 3 Results

Out of 486 patients, 159 (32.7%) experienced misdiagnosis (Table 1). Misdiagnosis cases were slightly higher among female than male group (33.3% vs. 32.4%). Misdiagnosis was significantly more frequent in the working-age group, accounting for 35.2% in those aged 20–39 years and 37.7% in those aged 40–59 years (*P* = 0.043). Misdiagnosis rates varied significantly by participant ethnicity (*P* = 0.002) and occupation (*P* = 0.007). The distribution of misdiagnosed cases was lower among the Miao ethnic group (24.2%) and non-farmers (19.2%). Those having primary education were less likely to be misdiagnosed compared to individual having above primary education level (30.2% vs. 36.6%).

**Table 1:** Background characteristics of leprosy patients having leprosy misdiagnosis.

Characteristics of participants	Leprosy misdiagnosis		
	No (%)	Yes (%)	*p*-value
Total	327 (67.3)	159 (32.7)	
Gender			0.840
Female	106 (66.7)	53 (33.3)	
Male	221 (67.6)	106 (32.4)	
Age (Years)			0.037
≤19	77 (79.4)	20 (20.6)	
20–39	147 (64.8)	80 (35.2)	
40–59	76 (62.3)	46 (37.7)	
≥ 60	27 (67.5)	13 (32.5)	
Ethnicity			0.002
Han	52 (56.5)	40 (43.5)	
Miao	144 (75.8)	46 (24.2)	
Zhuang	90 (60.8)	58 (39.2)	
Others	41 (73.2)	15 (26.8)	
Occupation			0.007
Farmer	268 (64.9)	145 (35.1)	
Non—farmer	59 (80.8)	14 (19.2)	
Education			0.137
Primary	206 (69.8)	89 (30.2)	
Above primary	121 (63.4)	70 (36.6)	
Leprosy classification			0.024
Tuberculoid	38 (80.9)	9 (19.1)	
Borderline tuberculoid	110 (73.3)	40 (26.7)	
Mid-borderline	19 (54.3)	16 (45.7)	
Borderline lepromatous	140 (62.8)	83 (37.2)	
Lepromatous	20 (64.5)	11 (35.5)	
Blood test			
Albumin level			0.432
Normal	236 (66.3)	120 (33.7)	
Low	20 (62.5)	12 (37.5)	
High	71 (72.4)	27 (27.6)	
Alanine aminotransferase level (ALT)			0.202
Normal	296 (68.5)	136 (31.5)	
Low	15 (62.5)	9 (37.5)	
High	16 (53.3)	14 (46.7)	
Aspartate aminotransferase level			0.213
Normal	269 (69.2)	120 (30.8)	
Low	12 (60)	8 (40)	
High	46 (59.7)	31 (40.3)	
Creatinine level			0.615
Normal	248 (68.5)	114 (31.5)	
Low	18 (64.3)	10 (35.7)	
High	61 (63.5)	35 (36.5)	
Eye-hand-foot score			0.505
No disability	256 (68.6)	117 (31.4)	
Mild disability	34 (61.8)	21 (38.2)	
Severe disability	37 (63.8)	21 (36.2)	
History of recurrence			0.063
No	297 (66.1)	152 (33.9)	
Yes	30 (81.1)	7 (18.9)	
Mode of detection			< 0.001
Investigation	58 (76.3)	18 (23.7)	
Contact tracing	73 (88)	10 (12)	
Dermatology	81 (48.8)	85 (51.2)	
Report	115 (71.4)	46 (28.6)	
Source of infection			< 0.001
Family	193 (77.2)	57 (22.8)	
Outside family	39 (58.2)	28 (41.8)	
Unknown	95 (56.2)	74 (43.8)	
Leprosy reaction			< 0.001
No	274 (71.7)	108 (28.3)	
Yes	53 (51)	51 (49)	
Presence of skin lesion			< 0.001
0–1	49 (89.1)	6 (10.9)	
2 to 5	59 (67.8)	28 (32.2)	
>5	219 (63.7)	125 (36.3)	
Taking preventive medicine			0.027
No	309 (66.3)	157 (33.7)	
Yes	18 (90)	2 (10)	
Village with multibacillary leprosy for 5 years			0.007
No	235 (64)	132 (36)	
15.6-7.4,-14.3242ptYes	92 (77.3)	27 (22.7)	
Village with multibacillary leprosy for 6–10 years			0.035
No	278 (65.6)	146 (34.4)	
15.6-7.4,-14.3242ptYes	49 (79)	13 (21)	
Village with multibacillary leprosy for over 10 years			0.912
No	266 (67.2)	130 (32.8)	
Yes	61 (67.8)	29 (32.2)	
History of long travel			0.05
No	231 (70.2)	98 (29.8)	
Yes	96 (61.1)	61 (38.9)	
Household with active case			0.653
No	294 (67)	145 (33)	
Yes	33 (70.2)	14 (29.8)	
Household with cured case			< 0.001
No	208 (61.4)	131 (38.6)	
Yes	119 (81)	28 (19)	

Clinically, leprosy misdiagnosis was more likely observed in 45% of mid-borderline lepromatous cases (*P* = 0.024). The misdiagnosis cases were observed in 46.7% of patients with higher levels of alanine aminotransferase in blood tests. Similarly, misdiagnosis tended to increase with higher levels of blood test parameters (albumin, alanine aminotransferase, aspartate aminotransferase, and creatinine), although these associations were not statistically significant. There was also no statistically significant difference in misdiagnosis cases among individuals with various severity of eye-hand-foot.

Misdiagnosis was observed in 18.9% of individuals with a history of recurrent leprosy, whereas those without a recurrence (33.9%) were more likely to be misdiagnosed. Detection of misdiagnosis cases was higher in dermatology clinics (51.2%) compared with other modes of detection Patients with different sources of infection had significant differences in the detection of misdiagnosed cases. Patients having more than five skin lesions (36.3%) were more likely to be misdiagnosed. Individuals without leprosy reaction (49.0%), were less likely to be misdiagnosed. In contrast, those without taking preventive medicine (33.7%), those living in village without multibacillary leprosy for 5 (36.0%) or 6–10 years (34.4%) and those living with households who were not cured (38.6%) were also more likely to be misdiagnosed. No association with misdiagnosis was observed among individuals living in villages with multibacillary leprosy for over 10 years, those with a long travel history, or those with active household cases.

### 3.1 Feature selection

**Figure 1:**
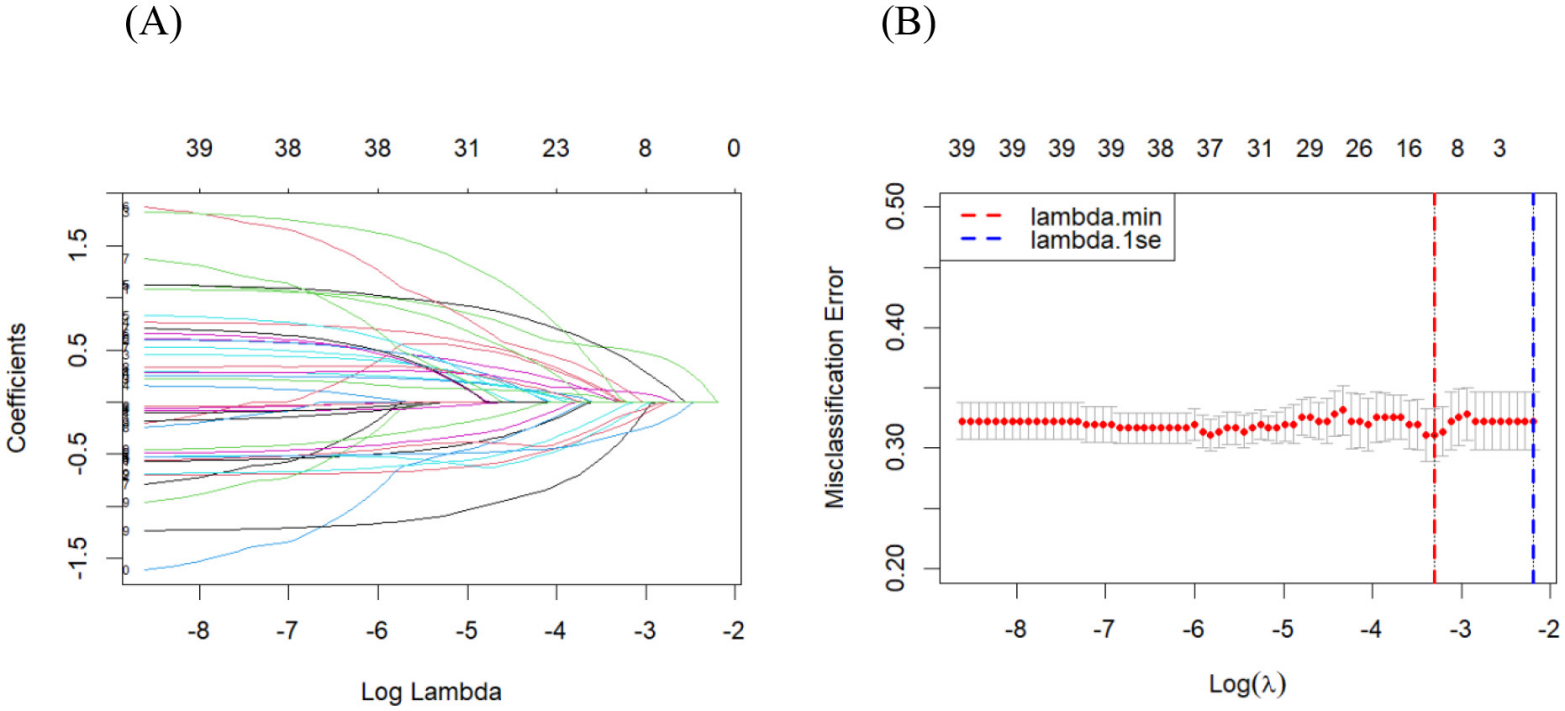
Feature selection using LASSO regression **(A)** regression coefficients of features and **(B)** relevant features in optimal λ.min value.

Figure 1A shows that as the optimal λ value approached between −4 and −3, the regression coefficients for most variables shrank toward zero. Figure 1B reveals that 19 variables were selected as relevant features at the optimal λ.min value indicated by the red vertical line, which minimizes the misclassification error and maximizes the predictive performance.

Patients with significant predictors included sociodemographic factors (gender, age group, ethnicity, occupation, and education), clinical characteristics (leprosy classification, albumin, aspartate aminotransferase level, alanine aminotransferase level, creatinine levels, history of recurrence, presence of leprosy reaction, and the number of skin lesions, preventive medication), and epidemiological/environmental factors (mode of detection, source of infection, village multibacillary status for 5 years and 6–10 years, and household exposure to active or cured cases).

### 3.2 Model performance

**Figure 2:**
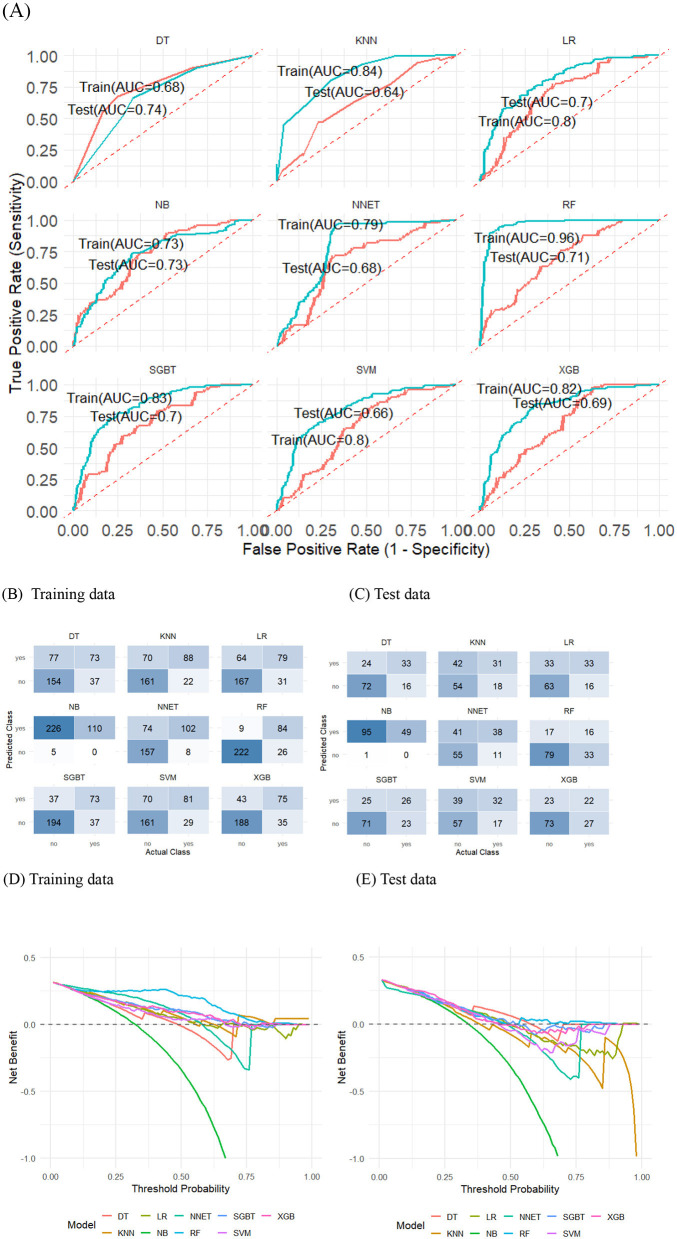
Model evaluation and validation in the training and test data using AUC **(A)**, confusion matrix of predicted and actual values (**B, C**, respectively) and decision curve analysis (**D, E**, respectively).

In Figure 2A, the ML models demonstrated varying performance levels based on their AUC scores in train and test data sets. In the training dataset, SGBT, XGB, LR, and NNET demonstrated high AUC values with excellent discrimination power, achieving 0.83, 0.82, 0.80, and 0.79, respectively. However, their performance declined by nearly 10% on the test data, with SGBT dropping to 0.70, XGB to 0.69, LR to 0.70, and NNET to 0.68, indicating moderate to modest reductions in generalization. In contrast, NB and DT exhibited lower AUC values with acceptable discrimination in the training data at 0.73 and 0.68, respectively, yet remained remarkably stable across datasets. Meanwhile, RF, KNN, and SVM achieved relatively high AUC values with excellent discrimination in the training data, recording 0.96, 0.84, and 0.80, respectively, but suffered performance declines of nearly 15% on the test data. RF experienced the largest drop to 0.71, suggesting severe overfitting, while KNN fell to 0.64 and SVM declined to 0.66, both indicating limited generalizability and poor robustness on unseen data.

Table 2 shows the comparison of accuracy, sensitivity, specificity, precision and F1 of each model in train and test data. All models' accuracy ranged between 0.34 and 0.90 in train data and between 0.34 and 0.72 in test data. In the training dataset, NNET achieved an accuracy of 0.76, which was lower than RF (0.90) and SGBT (0.78). The performance of NNET was characterized by high sensitivity (0.93) and moderate specificity (0.68). In contrast, the higher accuracy observed in RF was primarily attributable to its greater specificity (0.96), despite a lower sensitivity (0.76). Similarly, SGBT demonstrated higher specificity (0.84) but lower sensitivity (0.66) compared with NNET. In the test dataset, NNET's accuracy decreased to 0.64, remaining lower than DT (0.72) and slightly below SGBT, LR, RF, and XGB (each 0.66). The higher accuracy observed in these models except NNET was largely attributable to greater specificity (0.66–0.82) compared with NNET (0.57), but this was accompanied by reduced sensitivity (0.33–0.67). In contrast, NNET demonstrated a more balanced performance, achieving higher sensitivity (0.78) despite moderate specificity.

**Table 2:** Model performances of the training and test data.

Model	Accuracy	Sensitivity	Specificity	Precision	F1
Train					
DT	0.67	0.66	0.67	0.49	0.56
NB	0.34	1.00	0.02	0.33	0.49
SGBT	0.78	0.66	0.84	0.66	0.66
KNN	0.73	0.80	0.70	0.56	0.66
NNET	0.76	0.93	0.68	0.58	0.71
SVM	0.71	0.74	0.70	0.54	0.62
LR	0.72	0.72	0.72	0.55	0.62
RF	0.90	0.76	0.96	0.90	0.83
XGB	0.77	0.68	0.81	0.64	0.66
Test					
DT	0.72	0.67	0.75	0.58	0.62
NB	0.34	1.00	0.01	0.34	0.51
SGBT	0.67	0.53	0.74	0.51	0.52
KNN	0.59	0.63	0.56	0.42	0.51
NNET	0.64	0.78	0.57	0.48	0.59
SVM	0.61	0.65	0.59	0.45	0.53
LR	0.66	0.67	0.66	0.50	0.57
RF	0.66	0.33	0.82	0.48	0.39
XGB	0.66	0.45	0.76	0.49	0.47

In the meantime, NNET maintained a precision of 0.58 on the training data and 0.48 on the test data. This indicates that when NNET predicts a misdiagnosis, it is correct nearly half the time on unseen data. On the training data, models such as RF, SGBT and XGB achieved precision values of 0.90, 0.66 and 0.64, respectively, which were higher than that of NNET. However, their precision dropped to 0.48, 0.51 and 0.49 on the test data, respectively, which corresponded to a drop in sensitivity for RF (from 0.76 in training to 0.33 in test), for SGBT (0.66 to 0.53) and for XGB (from 0.68 to 0.45).

NNET achieved the highest F1-scores overall, with 0.71 in the training dataset and 0.59 in the test dataset. Although RF had a higher F1-score than NNET in the training data (0.83), its performance declined markedly in the test dataset (0.39), accompanied by low sensitivity (0.33). In contrast, NB had the lowest F1-score despite perfect sensitivity (1.00), as its specificity was nearly zero. Most other models had lower F1-scores than NNET in the training dataset, and their performance further deteriorated in the test dataset, with reduced sensitivity compared with NNET.

The confusion matrix is illustrated in Figure 2B for train and Figure 2C for test data. NNET model can predict higher number of misdiagnosed leprosy cases, 102 out of 110 cases in train data. It means that 92.7% of all misdiagnosed leprosy cases that were overlooked by health care providers would be able to detect via ML model. It was true for test data set, detecting 38 out of 49 misdiagnosed leprosy cases, 77.5%. In contrast, actual leprosy detection was lower with 157 out of 231 leprosy cases (67.9% case detection) in train and 55 out of 96 leprosy cases (57.2% case detection) in test data than the remaining models. As a result, false positive cases refer to individuals without leprosy who are incorrectly classified as having leprosy disease will increase and burden to further diagnosis.

The net benefit analysis in Figures Figure 2D and Figure 2E demonstrated increasing clinical utility with lower probability thresholds. The balance between true positives and false positives yielded significant net benefit at reduced cutoff values. The optimal decision threshold would be established below 0.25 for both the training and test datasets to evaluate model performance.

**Figure 3:**
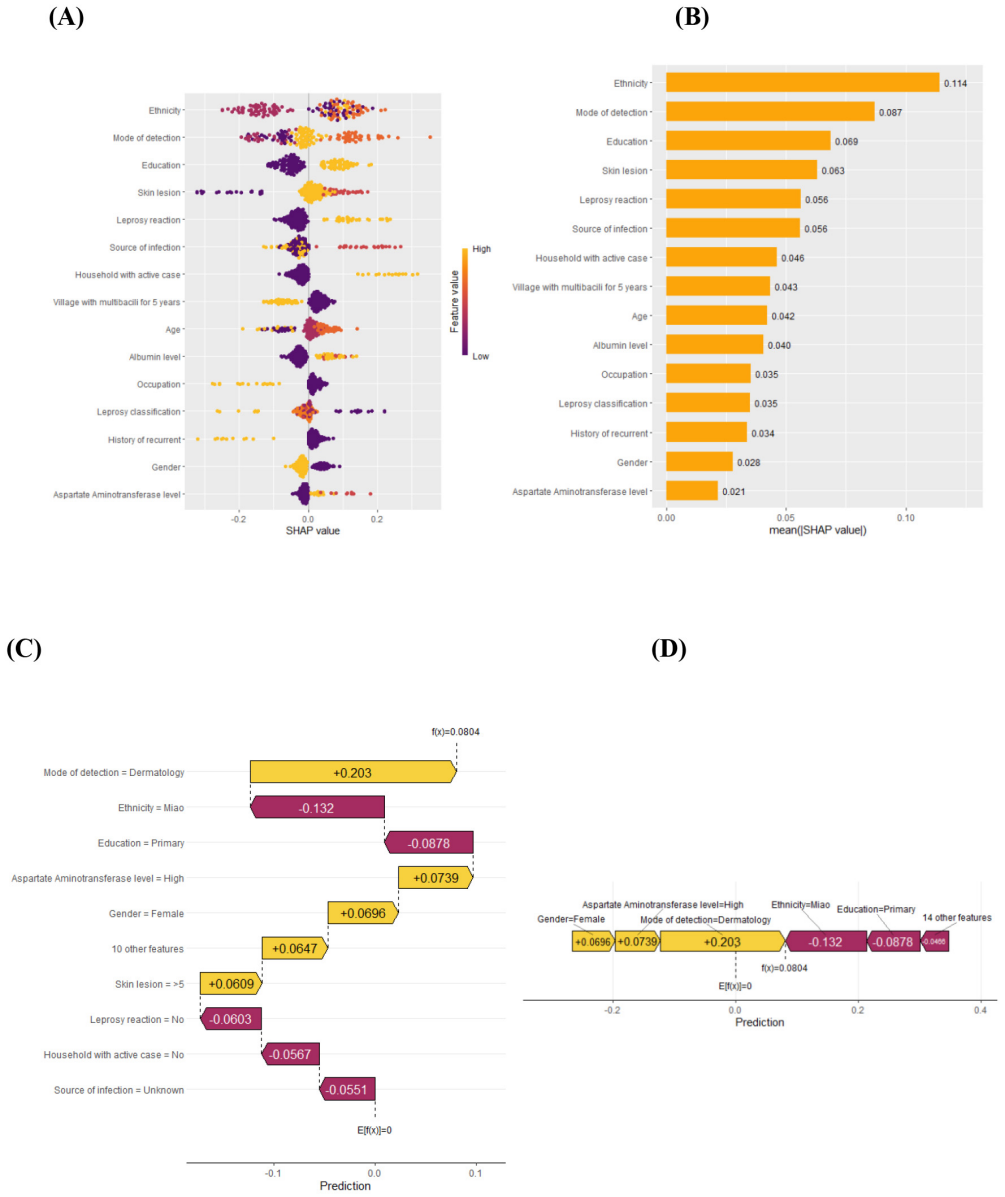
SHAP feature importance in beeswarm plot **(A)**, bar chart plot **(B)**, model explanation by the SHAP method in waterfall plot **(C)** and force plot **(D)**.

Figures 3A and 3B show that the common predictors that influence on misdiagnosis prediction were ethnicity (mean SHAP = 0.114) followed by mode of detection (0.087), education (0.069), skin lesion (0.063), leprosy reaction (0.056), and source of infection (0.056). The remaining predictors of household with active cases, villages with multibacillary for 5 years, age and albumin level, occupation, leprosy classification, history of recurrence, gender and aspartate aminotransferase level showed relatively smaller contributions (< 0.05).

Figures 3C and 3D also show that detection by a dermatology clinic, high aspartate aminotransferase level in the blood, being female, and having more than five skin lesions were the main positive contributors. Misdiagnosed cases were more likely to be detected in dermatology clinics compared to other settings. Female patients, those with an elevated aspartate aminotransferase level and those having five skin lesions were more likely to be misdiagnosed with other diseases rather than leprosy.

Conversely, being of Miao ethnicity, having only primary education, and presenting with no leprosy reaction, no household active case, and an unknown source of infection were main negative contributors that were less chance of being misdiagnosed.

## 4 Discussion

This study demonstrated the potential of machine learning (ML) to address the challenge of preventing a leprosy misdiagnosis. By analyzing a comprehensive set of clinical and epidemiological features, we developed predictive models that can identify patients at high risk of being misdiagnosed, thereby offering a pathway to prevent overlooking of the leprosy diseases by health care providers.

The performance of our machine learning models provides a realistic assessment of both the potential and the challenges of this approach. All models demonstrated lower sensitivity than NNET model. The misdiagnosis of leprosy was the condition that health care providers did not get diagnosis of the leprosy at the patient's initial visit to health center. Although the NNET model did not achieve perfect sensitivity, the results suggest that approximately 40–70% of potentially misdiagnosed leprosy cases by health care providers would be identified and flagged to health care providers for further evaluation as suspected leprosy (21).

In contrast, all models demonstrated higher specificity than the NNET model. However, lower specificity in the NNET model resulted in a greater number of false positives, meaning that some individuals without leprosy were incorrectly classified as having the leprosy disease. Given that leprosy can cause lifelong health consequences if not diagnosed early, an increased number of false positives at the screening stage may be acceptable in order to reduce delayed diagnosis (21).

Therefore, the selected NNET model demonstrates several strengths for scalability. Its higher sensitivity than other models make it suitable for early screening and integration into primary healthcare settings, where timely identification is critical. Additionally, NNET models can incorporate multiple clinical and demographic variables and adapt to complex, non-linear relationships, supporting broader applicability across diverse populations. However, limitations include lower specificity, potential overfitting, dependence on data quality, and reduced interpretability compared to traditional models, which may affect clinical trust and implementation. In addition, NNET offers moderate scalability for disease prediction due to its simple architecture, but it lags behind tree-based and deep models in handling massive datasets. Globally, ensembles like XGBoost provide superior large-scale performance, while deep networks excel with sufficient data and hardware (23).

Compared with models developed for other diseases globally, the performance of NNET in this study is consistent with prior evidence. Artificial neural networks have shown strong performance in tuberculosis detection, leprosy reaction risk prediction, adolescent obesity classification, and cognitive impairment prediction in chronic kidney disease (24–27). These findings suggest that while NNET models are robust and flexible across different clinical contexts, their optimal use often depends on balancing sensitivity and specificity according to the public health priority of the condition.

The interpretation of our models via SHAP analysis offers clinically actionable insights. The identification of mode of detection as one of the strongest predictors that correctly detected leprosy cases and misdiagnosed cases due to having well trained health staff. Previous findings suggest that human resources with capacity building could be most impactful when targeted at health care settings (3). Routine measurement of aspartate aminotransferase levels in the blood for all patients, regardless of gender, at the health center may provide potential benefits in achieving a more complete clinical assessment. The previous study in India shows that persistent higher level of aspartate aminotransferase levels in the blood among leprosy patients (28).

Our findings confirm the multifaceted nature of leprosy misdiagnosis. The significant associations with gender, ethnicity and education highlight specific demographic groups that may be particularly vulnerable to misdiagnosis that are possibly due to healthcare access barriers, stigma, or initial attribution of symptoms to occupational causes (3, 4). Furthermore, in epidemiology point of view, those with probably being low risk of getting leprosy such as presenting with fewer skin lesions, no leprosy reaction, no household active case, and an unknown source of infection were less chances of misdiagnosis. In another words, those with high risks are more likely to be misdiagnosis due to clinicians' low suspicion, atypical presentations, and unfamiliarity with the disease (4, 29).

Several limitations of this study must be acknowledged. First, SMOTE helps balance the data by creating new synthetic examples of the minority class, but it has some limitations. Because it generates artificial data, these new cases may not fully reflect real patients. If the original data contains noise or errors, SMOTE can amplify these issues by reproducing them (16). Second, the retrospective and single-source nature may introduce selection bias and limit the generalizability of the findings to other populations or healthcare systems. Third, the sample size, while sufficient for an initial exploration, is modest for ML modeling that potentially limits the complexity of the models we could train and their ultimate performance. Fourth, the models were built on data from already-diagnosed patients. Prospective validation in a real-world clinical setting where the model is applied to patients with undiagnosed skin lesions is the essential next step to prove its utility. Moreover, important predictive features, such specific descriptions of skin lesions or nerve thickening, and provider's data may not have been fully captured in the structured data used for this analysis. Finally, we could not evaluate misdiagnosed information from other record sources, and we could not perform cross-validation to assess the accuracy of clinical data.

Despite these limitations, this study established a strong foundation for the use of ML as a supportive tool in the fight against leprosy misdiagnosis. By moving beyond traditional risk factor analysis to build predictive models, we provide a scalable method to standardize clinical suspicion and assist frontline health workers. The future direction involves integrating such a model into a user-friendly clinical decision support system that is potentially a mobile or web-based application. This tool could help healthcare workers input patient characteristics and receive a validated risk score for leprosy that would prompt earlier referral and smear testing. By reducing misdiagnosis, such an innovation has the potential to avert disability, reduce transmission, and accelerate progress toward the global elimination of leprosy as a public health problem (30).

## 5 Conclusions

Machine learning is a tool that can be applied to clinical data to effectively identify leprosy patients who are at risk of misdiagnosis. Development of an ML-based support tool is needed to aid frontline healthcare providers for timely and accurate diagnoses, especially in settings where resources are limited. In order to meet global leprosy elimination goals, ML can be potential future implications requiring prospective validation to reduce misdiagnosis, prevent disabilities, and interrupt the transmission of leprosy.

## Data Availability

The original contributions presented in the study are included in the article/supplementary material, further inquiries can be directed to the corresponding author.
